# Plant species' origin predicts dominance and response to nutrient enrichment and herbivores in global grasslands

**DOI:** 10.1038/ncomms8710

**Published:** 2015-07-15

**Authors:** Eric W. Seabloom, Elizabeth T. Borer, Yvonne M. Buckley, Elsa E. Cleland, Kendi F. Davies, Jennifer Firn, W. Stanley Harpole, Yann Hautier, Eric M. Lind, Andrew S. MacDougall, John L. Orrock, Suzanne M. Prober, Peter B. Adler, T. Michael Anderson, Jonathan D. Bakker, Lori A. Biederman, Dana M. Blumenthal, Cynthia S. Brown, Lars A. Brudvig, Marc Cadotte, Chengjin Chu, Kathryn L. Cottingham, Michael J. Crawley, Ellen I. Damschen, Carla M. Dantonio, Nicole M. DeCrappeo, Guozhen Du, Philip A. Fay, Paul Frater, Daniel S. Gruner, Nicole Hagenah, Andy Hector, Helmut Hillebrand, Kirsten S. Hofmockel, Hope C. Humphries, Virginia L. Jin, Adam Kay, Kevin P. Kirkman, Julia A. Klein, Johannes M. H. Knops, Kimberly J. La Pierre, Laura Ladwig, John G. Lambrinos, Qi Li, Wei Li, Robin Marushia, Rebecca L. McCulley, Brett A. Melbourne, Charles E. Mitchell, Joslin L. Moore, John Morgan, Brent Mortensen, Lydia R. O'Halloran, David A. Pyke, Anita C. Risch, Mahesh Sankaran, Martin Schuetz, Anna Simonsen, Melinda D. Smith, Carly J. Stevens, Lauren Sullivan, Elizabeth Wolkovich, Peter D. Wragg, Justin Wright, Louie Yang

**Affiliations:** 1 Department of Ecology, Evolution, and Behavior, University of MN, St Paul, Minnesota 55108, USA.; 2 ARC Centre of Excellence for Environmental Decisions, School of Biological Sciences, The University of Queensland, Brisbane, Queensland 4072, Australia.; 3 School of Natural Sciences & Trinity Centre for Biodiversity Research, Zoology, Trinity College Dublin, Dublin 2, Ireland.; 4 Ecology, Behavior & Evolution Section, University of California, San Diego, La Jolla, California 92093, USA.; 5 Department of Ecology and Evolutionary Biology, University of Colorado, Boulder Colorado 80309, USA.; 6 School of Earth, Environmental and Biological Sciences, Queensland University of Technology, Brisbane, Queensland 4000, Australia.; 7 Department of Physiological Diversity, Helmholtz Center for Environmental Research – UFZ, Permoserstr. 15, 04318 Leipzig, Germany.; 8 German Centre for Integrative Biodiversity Research (iDiv) Halle-Jena-Leipzig, Deutscher Platz 5e, D-04103 Leipzig, Germany.; 9 Institute of Biology, Martin Luther University Halle-Wittenberg, Am Kirchtor 1, 06108 Halle (Saale), Germany.; 10 Ecology and Biodiversity Group, Department of Biology, Utrecht University, Padualaan 8, Utrecht 3584 CH, Netherlands.; 11 Department of Integrative Biology, University of Guelph, Guelph, Ontario, Canada N1G 2W1.; 12 Department of Zoology, University of Wisconsin, Madison, Wisconsin 53706, USA.; 13 CSIRO Land and Water Flagship, Wembley, Western Australia 6913, Australia.; 14 Department of Wildland Resources and the Ecology Center, Utah State University, Logan, Utah 84322, USA.; 15 Department of Biology, Wake Forest University, Winston-Salem, North Carolina 27109, USA.; 16 School of Environmental and Forest Sciences, University of Washington, Seattle, Washington 98195, USA.; 17 Department of Ecology, Evolution, and Organismal Biology, Iowa State University, Ames, Iowa 50011, USA.; 18 Rangeland Resources Research Unit, USDA Agricultural Research Service, Fort Collins, Colorado 80526, USA.; 19 Department of Bioagricultural Sciences and Pest Management, Colorado State University, Fort Collins, Colorado 80523, USA.; 20 Michigan State University, Department of Plant Biology, East Lansing, Michigan 48824, USA.; 21 University of Toronto Scarborough, Toronto, Ontario, Canada M1C 1A4.; 22 School of Life Sciences, Lanzhou University, Lanzhou 730000, China.; 23 Department of Biological Sciences, Dartmouth College, Hanover, New Hampshire 03755, USA.; 24 Department Biology, Imperial College London, Silwood Park, Ascot SL5 7PY, UK.; 25 Department of Ecology, Evolution and Marine Biology, University of California, Santa Barbara, California 93106, USA.; 26 U.S. Geological Survey Forest and Rangeland Ecosystem Science Center, Corvallis, Oregon 97331, USA.; 27 USDA-ARS Grassland Soil and Water Research Lab, Temple, Texas 76502, USA.; 28 Department of Entomology, University of Maryland, College Park Maryland 20742, USA.; 29 School of Life Sciences, University of KwaZulu-Natal, Scottsville, Pietermaritzburg 3209, South Africa.; 30 Department of Ecology, Evolutionary Biology, Yale University, New Haven, Connecticut 06520, USA.; 31 Department of Plant Sciences, University of Oxford OX1 3RB, UK.; 32 Carl-von-Ossietzky University, Institute for Chemistry and Biology of the Marine Environment, Wilhelmshaven 26382, Germany.; 33 INSTAAR, University of Colorado, Boulder, Colorado 80303, USA.; 34 USDA-ARS Agroecosystem Management Research Unit, Lincoln, Nebraska 68583, USA.; 35 Biology Department, University of St Thomas, Saint Paul, Minnesota 55105, USA.; 36 Department of Ecosystem Science & Sustainability, Colorado State University, Fort Collins, Colorado 80523, USA.; 37 School of Biological Sciences, University of Nebraska, Lincoln, Nebraska 68588, USA.; 38 Department of Integrative Biology, University of California, Berkeley, California 94720, USA.; 39 Department of Biology, University of New Mexico, Albuquerque, New Mexico 87131, USA.; 40 Department of Horticulture, Oregon State University, Corvallis, Oregon 97331, USA.; 41 Key Laboratory of Adaptation and Evolution of Plateau Biota, Northwest Institute of Plateau Biology, Chinese Academy of Sciences, Qinghai 810008, China.; 42 Yunnan Academy of Biodiversity, Southwest Forestry University, Kunming 650224, China.; 43 University of Toronto, Toronto, Ontario Canada M5S 2J7.; 44 Department of Plant & Soil Sciences, University of Kentucky, Lexington, Kentucky 40546, USA.; 45 Department of Biology, University of North Carolina, Chapel Hill North Carolina 27599, USA.; 46 Australian Research Centre for Urban Ecology, Melbourne, c/o School of Botany, University of Melbourne, Melbourne, Victoria 3010, Australia.; 47 School of Biological Sciences, Monash University, Melbourne, Victoria 3800, Australia.; 48 Department of Botany, La Trobe University, Bundoora, Melbourne, Victoria 3086, Australia.; 49 Department of Zoology, Oregon State University, Corvallis, Oregon 97331, USA.; 50 Swiss Federal Institute for Forest, Snow and Landscape Research, Birmensdorf 8903, Switzerland.; 51 National Centre for Biological Sciences, GKVK Campus, Bellary Road, Bangalore 560065, India.; 52 University of Toronto St George, Toronto, Ontario Canada M5S 2J7.; 53 Department of Biology, Colorado State University, Fort Collins, Colorado 80523, USA.; 54 Lancaster Environment Center, Lancaster University, Lancaster LA1 4YQ, UK.; 55 Biodiversity Research Centre, University of British Columbia, Vancouver, British Columbia, Canada V6T 1Z4.; 56 Department of Biology, Duke University, Box 90338, Durham North Carolina, USA.; 57 Department of Entomology, University of California, Davis, California 95616, USA.

## Abstract

Exotic species dominate many communities; however the functional significance of species' biogeographic origin remains highly contentious. This debate is fuelled in part by the lack of globally replicated, systematic data assessing the relationship between species provenance, function and response to perturbations. We examined the abundance of native and exotic plant species at 64 grasslands in 13 countries, and at a subset of the sites we experimentally tested native and exotic species responses to two fundamental drivers of invasion, mineral nutrient supplies and vertebrate herbivory. Exotic species are six times more likely to dominate communities than native species. Furthermore, while experimental nutrient addition increases the cover and richness of exotic species, nutrients decrease native diversity and cover. Native and exotic species also differ in their response to vertebrate consumer exclusion. These results suggest that species origin has functional significance, and that eutrophication will lead to increased exotic dominance in grasslands.

## Abstract (web summary)

It remains unclear whether exotic and native species are functionally different. Using a global grassland experiment, Seabloom *et al*. show that native and exotic species respond differently to two globally pervasive environmental changes, addition of mineral nutrients and alteration of herbivore density.

## Graphical abstract

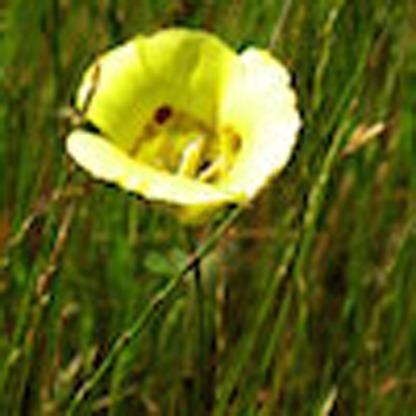

Conservation of biological diversity and ecosystem function in a dramatically changing world is a central issue for ecology in the coming decades^1^,^2^. In particular, as humans transport species to novel locations, the appropriate conservation responses to these novel species assemblages depends on our understanding of their effects on biological diversity and ecosystem function. Exotic species are abundant in many ecosystems^3^,^4^, and some island floras are almost completely composed of exotic species^4^. Economic costs associated with exotic species have been estimated at over $100 billion per year in the United States alone^5^, and there are well-documented cases of range reductions in native species and alteration of ecosystem functioning in response to invasion^3^,^4^. While exotic species currently dominate many communities^4^, it remains highly contentious whether exotic species function differently from the native, resident species^6^,^7^,^8^,^9^. The difficulty in separating these perspectives is exacerbated by a lack of globally replicated, systematic assessments of whether species origin is informative for predicting species' responses or function. Our current lack of data leaves a significant knowledge gap: we do not know how frequently species introduced by humans (exotic species) are functionally distinct from native species^6^,^7^,^8^,^10^,^11^.

Grouping species based on their origin or provenance (for example, native versus exotic species) may be meaningful if there are functional differences between native species and exotic species^6^,^7^,^10^,^11^,^12^,^13^,^14^,^15^,^16^. Functional differences between sympatric species of different origins can reflect processes acting over evolutionary time to create suites of species with unique traits that enable them to exploit empty niches in their introduced range (for example, the empty niche and the novel weapons hypotheses)^17^,^18^,^19^,^20^. For example, many invasive grassland plants have had a long association with intensive human agriculture and grazing and may be intentionally introduced or subjected to selective breeding programs^21^,^22^,^23^,^24^,^25^. Other species adapted to human-dominated landscapes typified by increased grazing pressure^21^,^25^ and increased nutrient supplies^22^,^23^ may be more likely to be unintentionally introduced because of their close association with human endeavours^24^. Furthermore, because species introductions and other anthropogenic changes are occurring simultaneously, understanding the impacts of exotic species requires understanding not only how native and exotic species differ in present-day ecosystems, but also how they differ in their responses to anthropogenic changes^6^,^26^. If exotic species differ from native species in their response to anthropogenic change, then species origin is clearly relevant for management.

On the other hand, exotic species at most sites are phylogenetically and functionally diverse, and grouping species based on their origin may not predict functional differences^8^,^13^. In this case, the distinction between native and exotic species may be uninformative for management of ecological function^13^. To date, there have been no globally replicated studies that use consistent methods to measure native and exotic abundance in response to experimental perturbations, and, in spite of ongoing debate in the literature^8^,^9^, we cannot currently resolve questions about the relevance of species origin.

We address this knowledge gap by evaluating whether native and exotic species differ in their abundance distributions and responses to nutrients and grazing by conducting a standardized experiment within grassland ecosystems in 13 countries on four continents (Supplementary Fig. 1 and Supplementary Table 1). Grasslands are a globally important biome that account for about one third of both terrestrial net primary production and ice-free land mass^27^ and are the most endangered of terrestrial ecosystems due to extensive conversion to human-dominated land uses^28^,^29^. Grasslands are also well suited for studying biological invasions, because many are highly invaded^21^,^24^, and changes in human land use, altered nutrient supplies, and altered consumer pressure have been suggested as drivers of invasion in the world's grasslands^21^,^25^,^30^,^31^ and in ecosystems generally^22^,^32^.

We examined changes in both the species richness and cover of native and exotic species because species richness measures the balance between colonization and extinction, whereas relative abundance provides insight into functional significance^31^,^33^,^34^. We test for differences between native and exotic species using both observational and experimental approaches. First, because dominant species typically have the greatest impact on essential ecosystem functions such as biomass production and nutrient cycling^33^, we test for differences in the abundance distributions of native and exotic species in an observational study conducted at 64 sites in 13 countries on 6 continents. A biased representation of functional traits in the exotic community could generate different responses by native and exotic species to changes in herbivory levels or nutrient supplies^10^,^11^,^13^,^21^,^26^,^35^,^36^, so we test whether groups of species favoured by fertilization and grazing, such as annual plants and grasses^37^,^38^, are more frequent in the pool of exotic or native species^13^.

Finally, given that anthropogenic perturbation could mediate invasion if species respond differently to environmental change^6^,^26^, we use two globally replicated experiments to directly test whether native and exotic species respond differently to nutrient enrichment and exclusion of vertebrate herbivores. In the Consumer by Nutrient Experiment, we manipulated vertebrate consumer density and nutrient supply in a fully factorial design by adding fences and a mixture of nutrients containing nitrogen, phosphorus, potassium, and micronutrients (NPK) at 34 sites. In the Multiple Nutrient Experiment, we added a fully factorial combination of three nutrient treatments (N, P and K plus micronutrients) at 37 sites in the presence of vertebrate consumers (unfenced plots).

We find that exotic species are six times more likely to be highly dominant in plant communities, and that native and exotic species respond differently to the experimental manipulation of nutrient supplies and vertebrate consumers. Addition of mineral nutrients leads to declines in native cover and richness, and corresponding increases in exotic cover. Exclusion of vertebrate consumers leads to an overall increase in native cover across all sites. Effects of vertebrate exclusion on native richness are mediated through change in light availability, while vertebrate effects on exotic richness are mediated through change in plant biomass.

## Results

### Abundance of native and exotic species

**Figure 1:**
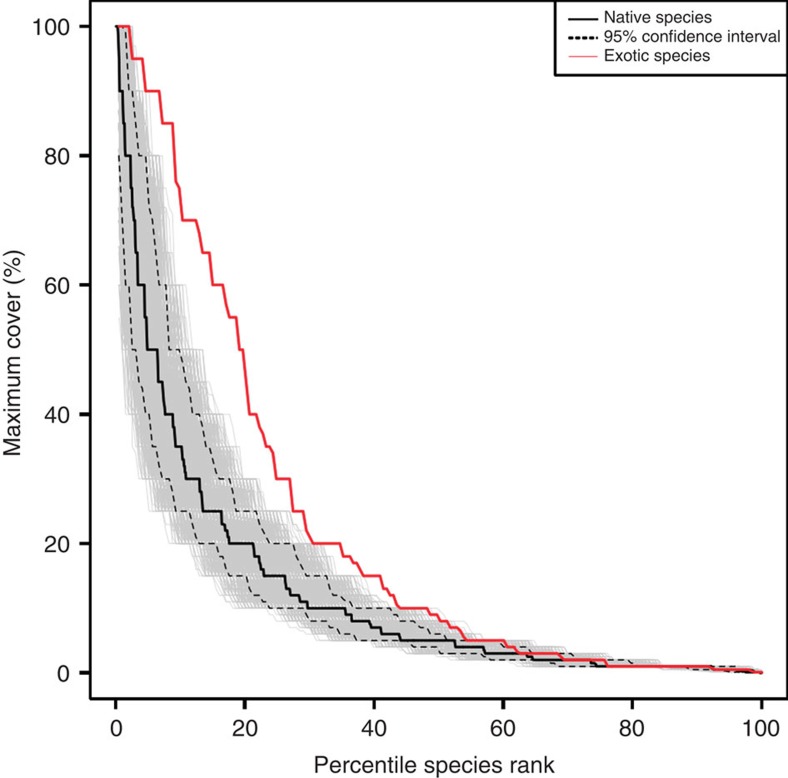
Rank abundance distribution of native and exotic species. Rank abundance plot showing maximal percent cover of native (*N*=1305) and exotic plant species (*N*=193) versus the relativized rank abundance, 100 *R*/*N* where R is the rank abundance of a native or introduced species (1 to *N*) and *N* is the total number of native or exotic species in the sample. The 95% confidence intervals (dashed lines) are calculated using 10,000 random bootstrap samples of 193 native species (grey lines). In this way, the confidence intervals control for the differences in the number of native and exotic species in the total data set.

Observational data from 64 grasslands in 13 countries revealed distinctly different patterns of abundance among native (*N*=1305) and exotic species (*N*=193; Fig. 1). Most native (92%) and exotic (75%) species were found at only one or two sites. While we found that both exotic and native species can dominate a local ecosystem, exotic species were more likely to dominate grasslands globally compared with their native counterparts. Exotic species were six times more likely to have a maximum cover of 80% or more of a surveyed area than were native species (8.8% versus 1.5% of species, respectively) and four times more likely to have a maximum cover of at least 50% (18.7% versus 4.8% of species, respectively; Fig. 1). Native and exotic species also were composed of different types of species. Across all sites, exotic species had a higher percentage of annual species and grasses (56% annuals and 31% grasses) than native species (21% annuals and 19% grasses; Supplementary Table 2; *P*<0.001; *n*=1642; *χ*^2^ test).

### Response of native and exotic species to nutrients and consumers

**Figure 2:**
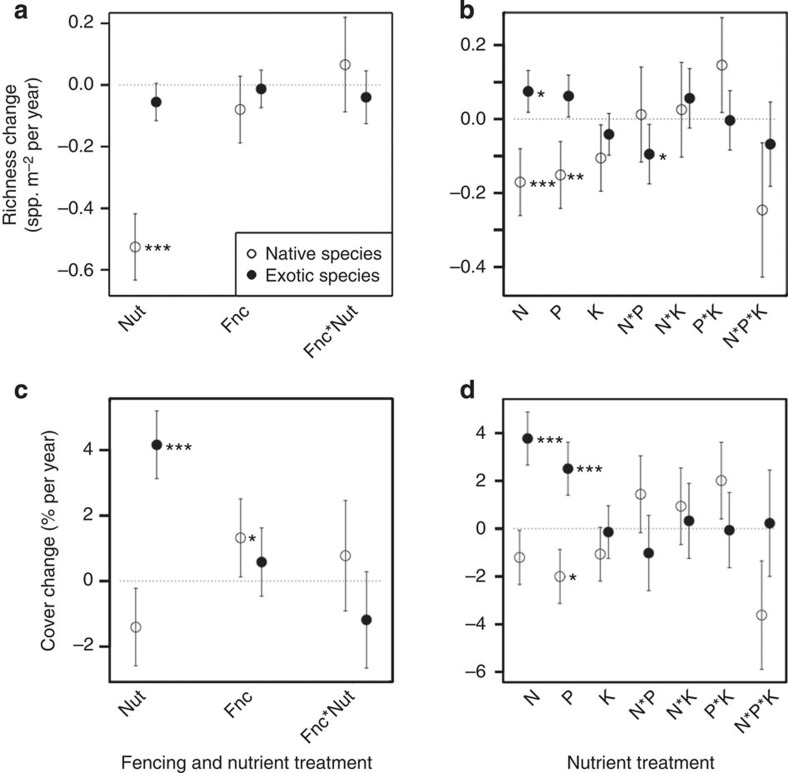
Nutrient and consumer effects on native and exotic cover and richness. Effect of nutrient addition and consumer exclusion on cover and richness of native and exotic plants. The Consumer by Nutrient Experiment (**a** and **c**) is a factorial combination of nutrient addition (Nut) and consumer exclusion (Fnc) replicated at 34 sites. The Multiple Nutrient Experiment (**b** and **d**) is a full factorial addition of nitrogen (N), phosphorus (P), and potassium with micronutrients (K) replicated at 37 sites. Plotted values are the estimated differences from the control of the change in cover or richness per year relative to pre-treatment sampling estimated using mixed-effects models. Interactions (for example, N*P, N*K, P*K, and N*P*K) test for additivity, with significant positive or negative values indicating super- or sub-additivity, respectively. Error bars are s.e. of the slope estimates. Slopes that are significantly different from zero are indicated as follows: ***P≤0.001, **P≤0.01 and *P≤0.05.

**Figure 3:**
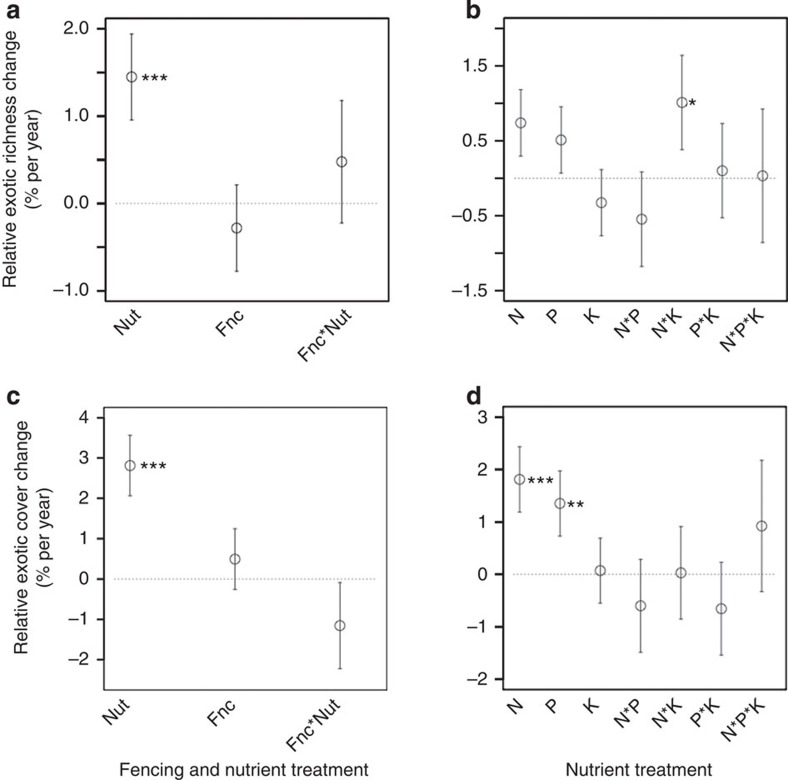
Nutrient and consumer effects on relative exotic cover and richness. Effect of nutrient addition and herbivore exclusion on the change in relative cover and richness of exotic plants. The Consumer by Nutrient Experiment (**a** and **c**) is a factorial combination of nutrient addition (Nut) and consumer exclusion (Fnc) replicated at 34 sites. The Multiple Nutrient Experiment (**b** and **d**) is a full factorial addition of nitrogen (N), phosphorus (P), and potassium with micronutrients (K) replicated at 37 sites. Plotted values are the treatment effects on the annual change in relative cover or richness of exotics per year estimated using mixed-effects models. Interactions (for example, N*P, N*K, P*K, and N*P*K) test for additivity, with significant positive or negative values indicating super- or sub-additivity, respectively. Exotic cover and richness was relativized by dividing by the total cover or richness in a plot multiplying by 100. Error bars are s.e. of the slope estimates. Slopes that are significantly different from zero are indicated as follows: ***P≤0.001, **P≤0.01 and *P≤0.05.

In the experimental manipulation of consumer density and nutrient supply conducted at 34 sites (Consumer by Nutrient Experiment), nutrient addition reduced native species richness but did not affect exotic richness. Native richness declined 0.5 species per year faster in fertilized than control plots, whereas there was no effect of any treatment on exotic richness (Supplementary Table 3; Fig. 2). Thus, the net effect of nutrients was an increase in the percent of exotic species in the fertilized plots (100 × exotic richness divided by total richness; Supplementary Table 3; Fig. 3). In contrast, fertilization left native cover unchanged, but led to a 3.5% per year increase in exotic cover relative to the control plots (Supplementary Table 5; Fig. 2). Therefore the addition of nutrients led to an increase in relative exotic cover in plots (100 × exotic cover divided by total cover; Supplementary Table 5; Fig. 3). Nutrient effects were independent of the pool sizes of soil nitrogen and phosphorus in the plots, as determined by pre-treatment sampling.

**Figure 4:**
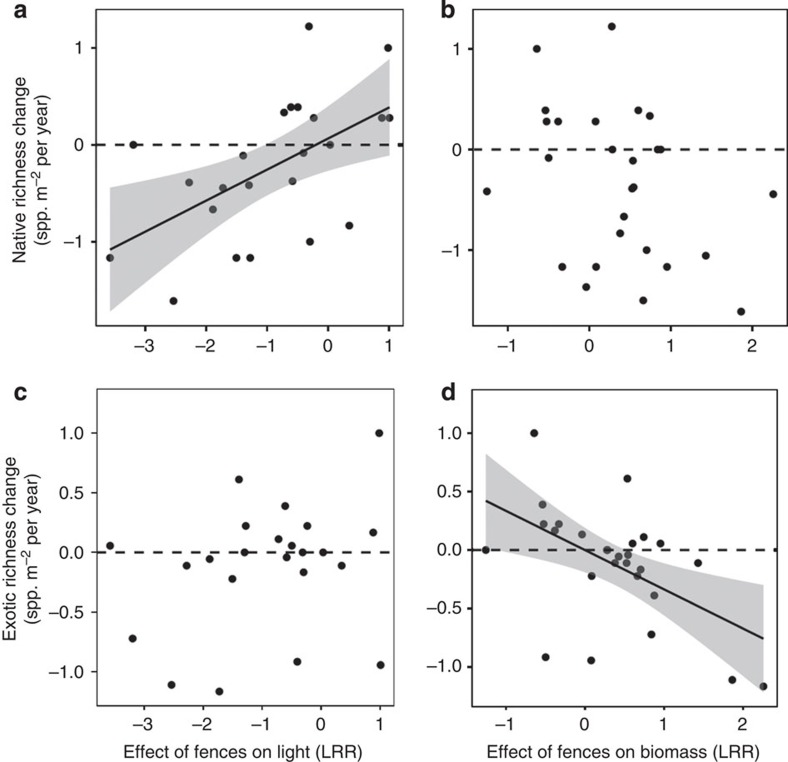
Effects of consumer fencing on native richness (a and b) exotic richness (c and d) ground-level light (a and c), and total biomass (b and d). Richness effects are measured as the difference from pre-treatment values. Light and biomass effects are presented as log ratios (difference of logged values) relative to pre-treatment values. All effects are shown after three years of treatment. Significant linear regressions (*P*<0.05) are shown with a 95% confidence interval, the grey shaded area.

Herbivore fencing led to 1.6% per year increase in the cover of native species; but there were no other significant effects of fencing. The intercepts in all models were not different from zero, indicating no net change across years in the control plots in the richness or cover of native or exotic species (Supplementary Table 3). Fencing effects on richness were highly variable across sites, and Borer *et al*.^39^ demonstrated that the strength of herbivore effects on total grassland diversity depends on the direct effects of herbivores on ground-level light and the indirect effects of herbivores on total biomass. Our current results extend this finding: we found that the relative importance of light and biomass differed for native and exotic species (Fig. 4). Excluding large herbivores led to declines in native richness at sites where the fences led to declines in ground-level light (*P*<0.001; Fig. 4). In contrast, herbivore fences decreased exotic richness at sites where they also increased biomass (*P*<0.001; Fig. 4). While total biomass and light are negatively correlated, the correlation is weak, and there are many cases with high biomass and high light and vice versa^39^. Thus, herbivores appear to influence exotic richness through their impacts on biomass, and native richness through their impacts on available light.

The factorial nutrient treatments in the Multiple Nutrient Experiment conducted at 37 sites further resolved the effects of specific nutrients on richness and cover. Nitrogen and phosphorus additions led to declines in native richness (Supplementary Table 4; Fig. 2). Nitrogen addition increased exotic richness when added alone, but there was a significant negative interaction with phosphorus, which may explain the lack of a significant effect in the Consumer by Nutrient Experiment where all nutrients were added in combination (Supplementary Table 4; Fig. 2). Nitrogen and phosphorus addition caused exotic cover to increase, and addition of phosphorus caused a decline in native cover (Supplementary Table 4; Fig. 2). Nutrient effects on exotic richness and cover were unaffected by the pre-treatment concentrations of N and P in the soils (*P*>0.05).

## Discussion

We found that exotic species were six times more likely to be highly abundant in control plots than were native species. An analysis of the subset of species for which we have data in both their native and exotic ranges, indicates that these exotic dominants are also highly abundant in their home ranges and that the potential to dominate as invaders is a characteristic of the species rather than advantage conferred on them by the loss of enemies or strong competitors^24^.

Native and exotic species responded differently to experimental nutrient additions and herbivore fencing across the broad range of nutrient supply, climatic and herbivore community gradients represented by the grassland sites in this study. Nutrient addition led to a decline in native richness and an increase in exotic cover. Removal of herbivores led to increased native cover. Fencing effects on native and exotic richness were associated with different drivers. In grasslands where herbivore fences decreased light, fences also decreased native richness. In contrast, exotic richness declined most strongly in grasslands where herbivore fences increased total biomass.

The consistent differences in the response of native and exotic species to nutrient addition and herbivore fences are particularly striking given the range of locations across six continents and the diverse assemblage of exotic species (193 species) covered by this study and the low overlap in species among sites; 92% of native species and 74% of exotic species occurred only at one or two sites and the most widespread species only occurs at 18% of the sites.

Some of these differences may reflect the coevolutionary history of exotic species with human agricultural areas that have high nutrient supply rates and heavy grazing^21^,^25^. There also were differences in the composition of the native and exotic communities that mirror those found in some regional floras^13^. Exotic species in our global-scale study were more likely than native species to have an annual life history and to be grasses, as has been demonstrated in regional-scale studies^13^. Annuals and grasses also have both been shown to be favoured by fertilization and grazing^37^,^38^. The differential responses of native and exotic species to nutrients also may be linked to systematic differences in functional traits associated with both high resource environments and exotic species (for example, foliar N content, specific leaf area, and photosynthetic capacity)^10^. Ultimately, it would be informative to isolate how much of the loss in native diversity is due to competitive interactions with the increasingly abundant exotic species, and how much of the decline is due to the characteristics of the native species themselves by crossing fertilization treatments with exotic species removal.

Our results suggest that differences between exotics and natives also extend to their interactions with herbivores and other consumers. Across all sites, native cover increased when vertebrates were excluded, but there was not a global effect of fencing on native or exotic richness. However, at sites where the presence of consumers increased ground-level light, they also increased the diversity of natives. Although, changes in light did not alter exotic diversity, exotic diversity declined where fences increased total biomass.

Thus, conservation of biotic interactions, such as herbivory, may be important for maintaining grassland plant communities that are richer in natives with fewer exotics, although the total cover of natives was higher in the absence of consumers. The current results suggest a tradeoff between conservation of native diversity and conservation of native dominance. Other studies have found that consumers can facilitate biological invasions^21^,^32^,^35^,^40^, but our results suggest that, across grasslands on six continents, consumers reduce exotic richness where they reduce total biomass. The lack of a main effect likely arises because herbivore effects on biomass vary among sites as a function of herbivore identity, provenance, density or feedbacks with the local abiotic environment^39^,^40^,^41^.

Two of the largest impacts of human activity on global ecosystems have been a rapid increase in nutrient loading and changes in consumer composition and abundance, both through extinctions and introductions^1^,^2^,^4^,^39^,^42^,^43^. Both of these impacts can alter invasion rates at a wide range of spatial scales^21^,^32^,^40^. The stronger response of exotic species to nitrogen and phosphorus suggests that adaptation to high nutrient conditions may be a trait shared by plants successful in human impacted systems^22^,^23^, possibly because of their association with agriculture^21^. This result is particularly worrisome as humans have dramatically increased the available pools of nitrogen and phosphorus^43^, and rates of addition appear to be continuing unabated^42^.

Previous studies have shown that nutrient addition can lead to local extinction and loss of diversity^39^,^44^,^45^, and so it is not surprising that native richness declined in response to nutrient addition. What is surprising is that native and exotic species differ so strongly in their responses to nutrients, and particularly that exotic richness was largely unaffected by nutrient addition. The lack of response in the exotic community suggests that different mechanisms regulate exotic and native diversity in grassland ecosystems^23^. The differing responses of native and exotic plant species to the effects of herbivory (altered biomass and ground-level light), also demonstrate that these plant provenance groups are functionally different^10^,^11^.

Despite the global homogenization of plant communities, only a small proportion of the total number of introduced species ultimately becomes established, and these species may comprise a functionally biased subset^11^. Our results suggest that successful exotic species, whether dominant or rare, are opportunistic species capable of persisting and increasing in response to increased nutrient supplies^10^,^22^,^23^ or altered rates of herbivory^21^,^32^,^35^,^40^. The most striking result of this study is that species of different provenance show consistent and different responses to nutrient addition under the wide range of abiotic and biotic conditions spanned by our global network of grassland sites. As a result of this differential response, continuing our history of global nutrient enrichment and modification of herbivore communities is likely to increase exotic dominance in many of the world's grasslands.

## Methods

### Sampling global grassland ecosytems

This work was conducted within the context of the Nutrient Network (NutNet), a globally replicated study of grassland ecosystems^46^. We collected observational data at 64 sites representing a wide range of herbaceous ecosystems including alpine tundra, montane meadows, mesic grasslands, savannas and annual grasslands. Sites also encompassed a wide range of environmental gradients including elevation (0–4,241 m), mean annual precipitation (211–2,072 mm per year), mean annual temperature (0–24 °C), latitude (38 °S–59 °N), and aboveground productivity (26–1,233 g m^−2^ per year). This set of sites also encompassed a wide range of soil conditions including site means for total nitrogen (267–11,980 p.p.m.), extractable P (8–229 p.p.m.), pH (4.0–8.3), sand (18–90%) and clay (1–44%). Plot scale (1 m^2^) diversity in the pre-treatment data ranged from 1.3 to 35.3 species m^−2^. Sites were selected without respect to the degree of invasion at the sites, and diversity and dominance of exotic species varied widely across these sites (0–98% of the species were exotic and 0–99% of the cover was composed of exotic species)^31^. We grouped our sites into regions that broadly correspond to the grazer coevolutionary regions of Milchunas *et al*.^25^ and Mack^21^. While overall exotic dominance varies among these regions as expected^31^, there were no consistent difference in fencing and nutrient treatment effects among these regions (*P*>0.05) and we do not include these regions in further analyses.

### Experimental manipulation of consumers and nutrients

At a subset of these sites, we conducted two parallel experiments, the Consumer by Nutrient Experiment (*n*=34) and the Multiple Nutrient Experiment (*n*=37) (Supplementary Fig. 1). Pre-treatment data were collected at all experimental sites used in the analyses in the year before the start of the treatments. Twenty-five sites started the experiment in 2007 with additional sites starting in subsequent years (Supplementary Table 1). Here we analyse data through 2013. Sampling and plot layout was the same at observational and experimental sites, a central feature of the Nutrient Network experimental design^46^.

Each experiment used a randomized block design typically with three replicate blocks per site (replication ranges from 1 to 6 blocks; Supplementary Table 1). The Consumer by Nutrient Experiment was a full factorial combination of nutrient addition (control or all nutrients) and consumer density (control or fenced) for a total of four treatments^46^. Fences, which were 2.1-m tall, were designed to exclude aboveground mammalian herbivores, including ungulates and lagomorphs (rabbits and hares). The top 1.2 m used five equally spaced rows of woven wire to prohibit large animals from jumping into the plots. The lower 0.9 m was 1 cm woven wire mesh with a 0.3 m outward-facing flange stapled to the ground to exclude digging animals; climbing mammals (for example, squirrels and mice) and fossorial mammals (for example, gophers) may have accessed fenced plots. Borer *et al*.^46^ provide a list of minor modifications made to the fencing protocol at some sites. The Multiple Nutrient Experiment was a full factorial combination of three nutrient addition treatments (N, P and other nutrients), each with two levels (control or added)^46^. Nutrient addition rates and sources were: 10 g N m^−2^ per year as time-release urea, 10 g P m^−2^ per year as triple super phosphate, 10 g K m^−2^ per year as potassium sulfate and 100 g m^−2^ per year of a micronutrient mix (6% Ca, 3% Mg, 12% S, 0.1% B, 1% Cu, 17% Fe, 2.5% Mn, 0.05% Mo and 1% Zn). N, P and K were applied annually; the micronutrient mix was applied once at the start of the study to avoid toxicity of largely immobile micronutrients. Note that ammonium nitrate was used in 2007 at some sites before switching to urea, however experiments at subset of the sites showed no effects of the nitrogen sources on biomass or richness^31^,^46^. While the nutrient addition rates were used at all sites, nutrient effects were unaffected by the pre-treatment concentrations of N and P in the soils (*P*>0.05).

### Sampling protocols

Sampling was conducted at peak biomass at each site in the year before the application of the experimental treatments, and in each year afterwards. Areal cover was estimated in a 1 m × 1 m quadrat located within the 5 m × 5 m area of each experimental plot. Cover was estimated independently for each species, so that summed cover can exceed 100% for multi-layered canopies, cover estimates have been effective at detecting species-level responses to treatments in these experiments^47^. Lead scientists at each site also provided data on each species at their sites including provenance (native or exotic), lifespan (annual, biennial, or perennial) and lifeform (grass, non-grass graminoid, forb, and woody plant). At some sites with distinct growth periods, cover was estimated at two periods and species were assigned the maximum cover across the two sampling dates. From pre-treatment data we calculated the maximum areal cover for each species across 1,923 1 m × 1 m quadrats (35,480 individual cover estimates) taken at the 64 grassland sites.

### Statistical analyses

All analyses were conducted using R version 3.0.2 (R Foundation for Statistical Computing, Vienna, Austria, 2013). We compared the maximum abundance distribution of native and exotic species using bootstrapped confidence intervals to demonstrate the likelihood of getting a curve similar to the suite of exotic species when making draws of 193 species from the native species pool (Fig. 1). This resampling controlled for the different numbers of native (*N*=1305) and exotic plant species (*N*=193) found in the overall data set. Similar results were found using bootstrapped confidence intervals for exotics, but this involves resampling smaller numbers of species (for examples, 100 natives and 100 exotics) to compare equal-sized pools of native and exotic species.

Our regression response metric (d*R*) was the rate of change in richness or cover per year relative to the pre-treatment values. Specifically, we calculated d*R=*(*R*_*t*_−*R*_0_)/*t* where *R*_*t*_ is the response metric after *t* years of treatment, *R*_0_ is the pre-treatment value of the response metric. We calculated the change in native richness, native summed cover, exotic richness, and exotic summed cover. Note that analysing richness and cover using raw values, proportional values (for example, exotic richness divided by total richness), arcsine square root-transformed proportions, or log response ratio (log[*R*_*t*_*/R*_0_]) did not qualitatively alter our conclusions. We also confirmed our conclusions by comparing the regression results to a permutation test, in which we compared the observed treatment effects to those calculated from 1,000 permutations of species provenance assignments within each site. There were not qualitative differences in the conclusions presented here and those from the permutation tests.

We analysed the cover and richness responses using mixed-effects models (nlme R package) in which site, block and treatment year (*t*, the number of years of treatments) were included as nested random effects^48^. Models were simplified using backward selection in two steps as in Crawley^48^. First, models were reduced by selecting the model with the lowest Akaike information criterion (AIC) using the stepAIC in the MASS R package. Second, all remaining higher-order terms were tested for significance (*P*<0.05) using likelihood ratio tests. We present full and reduced models in the Supplementary Tables.

For our experimental analyses, we did not include sites that had no recorded exotic species (largely Eurasian and African sites), as these zeros were uninformative. In addition, new NutNet sites were added since 2008, so we only had multiple years of data for 35 of the 37 sites that had exotic species present. We analysed the experimental data for years 2, 3, and 4 separately, and with the full data set with treatment year (1–5) included as a random effect. Because results were similar for all subsets of data, here we present results for the years 2–4 with year as a random effect, as this subset balances number of sites and having multiple years per site. Although we did not expect any consistent correlation across the globe due to differences among calendar years, we included year in initial models. However, year was not retained in any final model, and inclusion of this factor did not alter any of the conclusions or improve model fit.

We tested whether our results were sensitive to undercounting rare species by redoing all analyses using only species with >1% cover, and found removal of rare species did not change our conclusions. We also calculated summed native and exotic cover with all species and with the subsets of only species >1% cover, >5% cover, and >10% cover. All of these summed cover estimates for native and exotic species have correlation coefficients >0.96, indicating that there is little effect of the rare species on our estimates of summed cover (our measure of exotic and native dominance).

It is possible that the change in native or exotic diversity is constrained by the size of the species pool present at each site or by the proportion of the total species pool present in each sample^49^. We estimated the size of the relevant species pool by tallying all species found in 30 plots across 4 years of sampling (one pre-treatment year and three treatment years). The relative size of the native and exotic species pools varied from 2 to 95% exotic species. The site-level native species pool ranges from 1 to 72 species and the site-level exotic species pool ranges from 1 to 43 species. Individual quadrats contained from 3 to 50% of the site native species pool and 2 to 70% of the site exotic species pool. Thus, across all our sites, we have a wide range of combinations of native and exotic species pools as well as the percent of the species pool in each sample (quadrat). This variability allows us to test the impacts of species pool size on species responses. To do this, we added the following variables as covariates to our analysis of native and exotic species richness responses: number of exotic species at the site, number of native species at the site, mean proportion of the native species pool in an average quadrat and the mean proportion of the exotic species pool in an average quadrat. The addition of these covariates did not qualitatively change conclusions for any of analyses of changes in species richness among treatments.

## Additional information

**How to cite this article**: Seabloom, E.W. *et al*. Plant species' origin predicts dominance and response to nutrient enrichment and herbivores in global grasslands. *Nat. Commun.* 6:7710 doi: 10.1038/ncomms8710 (2015).

## Supplementary Material

Supplementary Information Supplementary Figure 1 and Supplementary Tables 1-7
