# Supplementary material for: Plant species' origin predicts dominance and response to nutrient enrichment and herbivores in global grasslands
Source: Nat Commun. 2015 Jul 15;6:7710. doi: 10.1038/ncomms8710 (PMC4518311; doi:10.1038/ncomms8710)
Supplement: Supplementary Information — Supplementary Figure 1 and Supplementary Tables 1-7 [file ncomms8710-s1.pdf]

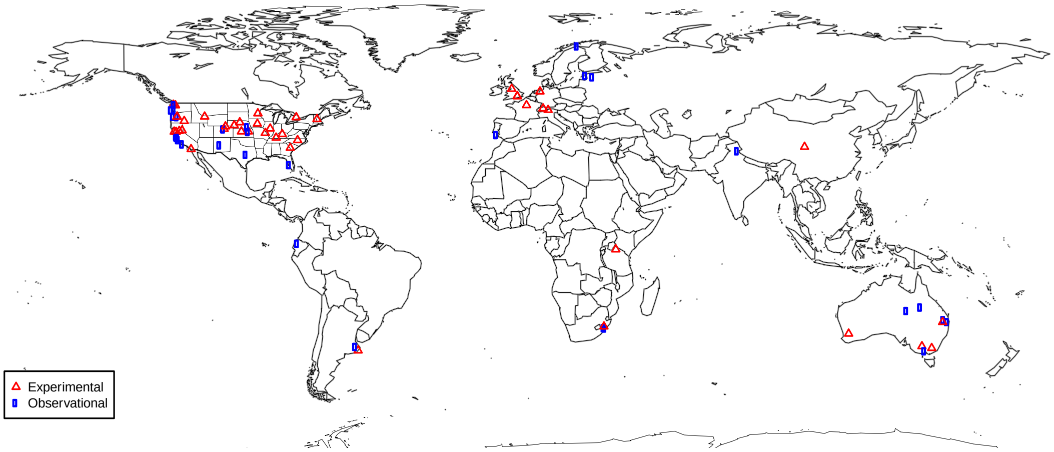

Supplementary Figure 1. Nutrient Network sites included in the current study.

Supplementary Table 1: Study sites included in analyses. Pretreatment observational data were collected at all sites. Subsequent fertilization and fencing were only conducted at experimental sites. Note that we only analyze experimental data from sites sampled for the first time before 2010.

ID	Site Name	Continent	Country	Ecosystem Type	Latitude	Longitude	Elevation (m)	Annual Precipitation (mm)	Study Type	Number Plots	First Sample Year	Site Species Richness	% Introduced Species
1	Serengeti	Africa	Tanzania	Savanna	-2.25	34.51	1536	854	Experimental	30	2008	46	0
2	Mt Gilboa	Africa	South Africa	Montane grassland	-29.28	30.29	1748	926	Experimental	30	2009	65	0
3	Summerveld	Africa	South Africa	Mesic grassland	-29.81	30.72	679	939	Experimental	30	2009	94	0
4	Ukulinga	Africa	South Africa	Mesic grassland	-29.67	30.4	842.5	880	Experimental	30	2009	93	2
5	Azi	Asia	China	Alpine grassland	33.67	101.87	3500	667	Experimental	30	2007	65	0
6	Kibber (Spiti)	Asia	India	Alpine grassland	32.32	78.01	4241	504	Experimental	30	2011	23	0
7	Bogong	Australia	Australia	Alpine grassland	-36.87	147.25	1760	1592	Experimental	30	2009	34	6
8	Bunya Mountains	Australia	Australia	Grassland	-26.89	151.61	0	871	Observational	30	2013	17	18
9	Burrawan	Australia	Australia	Semiarid grassland	-27.73	151.14	425	683	Experimental	30	2008	36	14
10	Derrimut	Australia	Australia	Semiarid grassland	-37.81	144.79	38	574	Observational	30	2007	19	47
11	Ethabuka (South Site)	Australia	Australia	Desert grassland	-23.64	138.4	104	211	Experimental	30	2013	6	0
12	Kinypanial	Australia	Australia	Semiarid grassland	-36.2	143.75	90	426	Experimental	30	2007	69	29
13	Mitchell grassland	Australia	Australia	Semiarid grassland	-22.48	143.32	242	425	Observational	10	2013	17	0
14	Mt. Caroline	Australia	Australia	Savanna	-31.78	117.61	285	330	Experimental	40	2008	47	28
15	Pinjarra Hills	Australia	Australia	Pasture	-27.53	152.92	38	1133	Experimental	30	2013	28	57
16	Fruebuel	Europe	Switzerland	Pasture	47.11	8.54	995	1355	Experimental	30	2008	27	11
17	Val Mustair	Europe	Switzerland	Alpine grassland	46.63	10.37	2320	1098	Experimental	30	2008	84	0
18	Papenburg	Europe	Germany	Old field	53.09	7.47	0.5	783	Experimental	10	2007	7	0

19	Elva	Europe	Estonia	Semiarid grassland	58.26	26.35	63.5	616	Observational	10	2012	51	0
20	Kirikukyla	Europe	Estonia	Calcareous grassland	58.71	23.8	7.5	601	Observational	30	2012	84	0
21	Companhia das Lezirias	Europe	Portugal	Annual grassland	38	-8	200	554	Experimental	31	2012	44	2
22	Heronsbrook (Silwood Park)	Europe	United Kingdom	Mesic grassland	51.41	-0.64	60	692	Experimental	30	2007	36	6
23	Lancaster	Europe	United Kingdom	Mesic grassland	53.99	-2.63	180	1322	Experimental	26	2008	16	0
24	Rookery (Silwood Park)	Europe	United Kingdom	Mesic grassland	51.41	-0.64	60	706	Experimental	30	2007	21	10
25	Koffler Scientific Reserve at Joker's Hill	North America	Canada	Pasture	44.02	-79.54	301	815	Experimental	36	2010	22	41
26	Cowichan	North America	Canada	Old field	48.46	-123.38	50	764	Experimental	30	2007	17	88
27	Hanover	North America	United States	Old field	43.42	-72.14	271	1033	Experimental	30	2007	36	58
28	Savannah River	North America	United States	Savanna	33.34	-81.65	71	1194	Experimental	20	2007	53	2
29	Duke Forest	North America	United States	Old field	36.01	-79.02	141	1163	Experimental	30	2007	36	31
30	Barta Brothers	North America	United States	Mixedgrass prairie	42.24	-99.65	767	597	Experimental	30	2007	42	2
31	Boulder South Campus	North America	United States	Shortgrass prairie	39.97	-105.23	1633	425	Experimental	20	2008	17	71
32	Chichaqua Bottoms	North America	United States	Tallgrass prairie	41.79	-93.39	275	855	Experimental	54	2009	58	14
33	Cedar Creek LTER	North America	United States	Tallgrass prairie	45.4	-93.2	270	750	Experimental	50	2007	59	20
34	Cedar Point Biological Station	North America	United States	Shortgrass prairie	41.2	-101.63	965	445	Experimental	60	2007	49	8
35	Hall's Prairie	North America	United States	Tallgrass prairie	36.87	-86.7	193.6	1282	Experimental	30	2007	21	5
36	Konza LTER	North America	United States	Tallgrass prairie	39.07	-96.58	440	877	Experimental	30	2007	53	2
37	KonzaN1B	North America	United States	Tallgrass prairie	39.08	-96.56	440	878	Observational	12	2007	68	6
38	Saline Experimental	North America	United States	Mixedgrass prairie	39.05	-99.1	440	607	Experimental	30	2008	61	11

	Range												
39	Sevilleta LTER	North America	United States	Desert grassland	34.36	-106.69	1600	252	Experimental	40	2007	37	0
40	Shortgrass Steppe LTER	North America	United States	Shortgrass prairie	40.82	-104.77	1650	365	Experimental	41	2007	21	5
41	Spindletop	North America	United States	Pasture	38.14	-84.5	271.3	1140	Experimental	30	2007	38	45
42	Temple	North America	United States	Tallgrass prairie	31.04	-97.35	184	871	Experimental	26	2007	98	4
43	Trelease	North America	United States	Tallgrass prairie	40.08	-88.83	200	982	Experimental	30	2008	19	16
44	Tyson	North America	United States	Old field	38.52	-90.56	169	997	Experimental	40	2007	60	10
45	Bunchgrass (Andrews LTER)	North America	United States	Montane grassland	44.28	-121.97	1318.083	1647	Experimental	30	2007	48	6
46	Buttercup (Andrews LTER)	North America	United States	Montane grassland	44.28	-121.96	1500	1718	Observational	30	2007	66	8
47	Hart Mountain	North America	United States	Shrub steppe	42.72	-119.5	1508	272	Experimental	30	2007	49	8
48	Lookout (Andrews LTER)	North America	United States	Montane grassland	44.21	-122.13	1500.492	1898	Experimental	30	2007	38	5
49	Niwot Ridge LTER	North America	United States	Alpine grassland	39.99	-105.38	3050	439	Observational	40	2007	43	0
50	Sagehen Creek UCNRS	North America	United States	Montane grassland	39.43	-120.24	1920	882	Experimental	30	2007	46	0
51	Sheep Experimental Station	North America	United States	Shrub steppe	44.24	-112.2	910	262	Experimental	40	2007	83	16
52	American Camp	North America	United States	Mesic grassland	48.47	-123.01	41	557	Observational	30	2007	26	73
53	Elliott Chaparral	North America	United States	Annual grassland	32.88	-117.05	200	331	Experimental	30	2008	43	47
54	Finley NWR	North America	United States	Mesic grassland	44.41	-123.28	68	1104	Observational	19	2007	64	45
55	Glacial Heritage	North America	United States	Mesic grassland	46.87	-123.03	33	1311	Observational	30	2007	17	65
56	Hastings UCNRS	North America	United States	Annual grassland	36.2	-121.55	750	702	Observational	30	2007	32	41
57	Hopland REC	North America	United States	Annual grassland	39.01	-123.06	598.302	1127	Experimental	30	2007	114	40
58	Jasper Ridge	North	United	Annual	37.41	-122.24	120	592	Observational	30	2007	34	74

	Biological Preserve	America	States	grassland									
59	Leadbetter Point	North America	United States	Salt marsh	46.61	-124.05	2	2072	Observational	30	2007	13	0
60	Mclaughlin UCNRS	North America	United States	Annual grassland	38.86	-122.41	641.801	867	Experimental	30	2007	62	42
61	Sedgwick Reserve UCNRS	North America	United States	Annual grassland	34.7	-120.02	550	521	Experimental	30	2007	41	41
62	Sierra Foothills REC	North America	United States	Annual grassland	39.24	-121.28	197.195	935	Experimental	50	2007	96	42
63	Smith Prairie	North America	United States	Mesic grassland	48.21	-122.62	62	597	Experimental	30	2007	53	81
64	Mar Chiquita	South America	Argentina	Grassland	-37.72	-57.42	6	838	Experimental	30	2011	38	16

Supplementary Table 2. Percent of native and exotic species in various lifespan and functional grouping. X^2 test for differences in lifespan by provenance and functional group by provenance distributions is based on 276 exotic and 1366 native species for which we had data on lifespan and functional group classifications. Forbs are herbaceous dicots, Graminoids are species in the Poales excluding the Poaceae, Grasses are members of Poaceae, and Woody Plants can be from any family.

	**Lifespan**			
	**Annual**	**Biennial**	**Indeterminate**	**Perennial**
Exotic	56	9	3	33
Native	21	3	3	74

$X^2 = 191.17$, $p < 0.001$

	**Functional Group**			
	**Forbs**	**Graminoids**	**Grasses**	**Woody Plants**
Exotic	67	0	31	2
Native	72	4	19	5

$X^2 = 29.59$, $p < 0.001$

Supplementary Table 3. Reduced mixed-effects regression models examining the change in native and exotic richness and cover on a factorial combination of fertilization and fencing (Consumer by Nutrient Experiment). Relative exotic richness and cover are normalized by dividing by the total richness and cover respectively and multiplying by 100. Site, block, plot, and years of treatment application are included as random effects in each regression model. Slopes that are significantly different from zero are indicated as follows: *** ($p \leq 0.001$), ** ($p \leq 0.01$), and * ($p \leq 0.05$).

**Response**	**Treatments**	**Estimate**	**SE**	**DF**	**t**	**p**	
Native Richness	Intercept	-0.105	0.106	722	-0.996	0.320	
	Nutrient Addition	-0.493	0.077	302	-6.437	0.000	***
Exotic Richness	Intercept	-0.075	0.069	722	-1.092	0.275	
Relative Exotic Richness	Intercept	0.027	0.392	722	0.069	0.945	
	Nutrient Addition	1.684	0.350	302	4.806	0.000	***
Native Cover	Intercept	3.230	1.686	722	1.916	0.056	
	Fencing	1.695	0.845	302	2.005	0.046	*
Exotic Cover	Intercept	-1.242	1.868	722	-0.665	0.506	
	Nutrient Addition	3.574	0.735	302	4.859	0.000	***
Relative Exotic Cover	Intercept	-0.765	0.896	722	-0.854	0.393	
	Nutrient Addition	2.243	0.534	302	4.196	0.000	***

Supplementary Table 4. Reduced mixed-effects regression models examining the change in exotic richness and cover on a factorial combination phosphorus (P), nitrogen (N), and potassium with micronutrients (K)(Multiple Nutrient Experiment). Relative exotic richness and cover are normalized by dividing by the total richness and cover respectively and multiplying by 100. Site, block, plot, and years of treatment application are included as nested random effects in each regression model. Slopes that are significantly different from zero are indicated as follows: *** ($p \leq 0.001$), ** ($p \leq 0.01$), and * ($p \leq 0.05$).

Response	Treatments	Estimate	SE	DF	t	p	
Native Richness	Intercept	-0.216937	0.131	1621.000	-2	0.099	
	N addition	-0.2147578	0.046	803.000	-5	0.000	***
	P addition	-0.1345677	0.046	803.000	-3	0.003	**
Exotic Richness	Intercept	-0.06725609	0.058	1621.000	-1	0.250	
	N addition	0.10274036	0.040	802.000	3	0.011	*
	P addition	0.0598623	0.040	802.000	1	0.138	
	N*P	-0.12849762	0.057	802.000	-2	0.025	*
Relative Exotic Richness	Intercept	0.3769764	0.348	1621.000	1	0.278	
	N addition	0.475043	0.316	802.000	2	0.133	
	P addition	-0.2652935	0.315	802.000	-1	0.400	
	N*K	1.0197065	0.447	802.000	2	0.023	*
Native Cover	Intercept	2.442504	1.881	1621.000	1	0.194	
	P addition	-1.204296	0.570	804.000	-2	0.035	*
Exotic Cover	Intercept	-1.773171	1.357	1621.000	-1	0.192	
	N addition	3.490205	0.557	803.000	6	0.000	***
	P addition	2.027408	0.557	803.000	4	0.000	***
Relative Exotic Cover	Intercept	-0.8015939	0.750	1621.000	-1	0.286	
	N addition	1.76112	0.313	803.000	6	0.000	***
	P addition	0.9552652	0.313	803.000	3	0.002	**

Supplementary Table 5. Full mixed-effects regression models examining the change in native and exotic richness and cover on a factorial combination of fertilization and fencing (Consumer by Nutrient Experiment). Relative exotic richness and cover are normalized by dividing by the total richness and cover respectively and multiplying by 100. Site, block, plot, and years of treatment application are included as random effects in each regression model. Slopes that are significantly different from zero are indicated as follows: *** ($p \leq 0.001$), ** ($p \leq 0.01$), and * ($p \leq 0.05$).

Response	Treatments	Estimate	SE	DF	t	p	
Native Richness	Intercept	-0.066	0.118	722	-0.562	0.574	
	Fencing	-0.080	0.108	300	-0.734	0.464	
	Nutrient Addition	-0.525	0.108	300	-4.875	0.000	***
	Fencing*Nutrient Addition	0.066	0.154	300	0.428	0.669	
Exotic Richness	Intercept	-0.031	0.078	722	-0.402	0.688	
	Fencing	-0.013	0.061	300	-0.211	0.833	
	Nutrient Addition	-0.055	0.061	300	-0.911	0.363	
	Fencing*Nutrient Addition	-0.040	0.086	300	-0.462	0.644	
Relative Exotic Richness	Intercept	0.163	0.461	722	0.354	0.723	
	Fencing	-0.280	0.495	300	-0.566	0.572	
	Nutrient Addition	1.449	0.493	300	2.941	0.004	**
	Fencing*Nutrient Addition	0.478	0.702	300	0.681	0.496	
Native Cover	Intercept	3.919	1.783	722	2.198	0.028	*
	Fencing	1.320	1.191	300	1.108	0.269	
	Nutrient Addition	-1.403	1.184	300	-1.184	0.237	
	Fencing*Nutrient Addition	0.775	1.687	300	0.459	0.646	
Exotic Cover	Intercept	-1.525	1.937	722	-0.787	0.432	
	Fencing	0.582	1.040	300	0.560	0.576	
	Nutrient Addition	4.156	1.034	300	4.019	0.000	***
	Fencing*Nutrient Addition	-1.181	1.473	300	-0.801	0.424	
Relative Exotic Cover	Intercept	-1.004	0.969	722	-1.037	0.300	
	Fencing	0.493	0.755	300	0.652	0.515	
	Nutrient Addition	2.814	0.751	300	3.747	0.000	***
	Fencing*Nutrient Addition	-1.156	1.070	300	-1.081	0.281	

Supplementary Table 6. Full mixed-effects regression models examining the change in exotic richness and cover on a factorial combination phosphorus (P), nitrogen (N), potassium with micronutrients (K)(Multiple Nutrient Experiment). Relative exotic richness and cover are normalized by dividing by the total richness and cover respectively and multiplying by 100. Site, block, plot, and years of treatment application are included as nested random effects in each regression model. Slopes that are significantly different from zero are indicated as follows: *** ($p \leq 0.001$), ** ($p \leq 0.01$), and * ($p \leq 0.05$).

**Response**	**Treatments**	**Estimate**	**SE**	**DF**	**t**	**p**	
Native Richness	Intercept	-0.193	0.140	1621	-1.377	0.169	
	N addition	-0.171	0.090	798	-1.886	0.060	
	P addition	-0.151	0.090	798	-1.672	0.095	
	K & Micronut. Addition	-0.105	0.090	798	-1.172	0.242	
	N*P	0.012	0.129	798	0.094	0.925	
	N*K	0.026	0.128	798	0.199	0.843	
	P*K	0.146	0.128	798	1.138	0.256	
	N*P*K	-0.246	0.182	798	-1.353	0.177	
Exotic Richness	Intercept	-0.047	0.065	1621	-0.731	0.465	
	N addition	0.075	0.057	798	1.323	0.186	
	P addition	0.062	0.057	798	1.101	0.271	
	K & Micronut. Addition	-0.041	0.056	798	-0.730	0.465	
	N*P	-0.095	0.081	798	-1.174	0.241	
	N*K	0.056	0.081	798	0.699	0.485	
	P*K	-0.004	0.081	798	-0.045	0.965	
	N*P*K	-0.068	0.114	798	-0.595	0.552	
Relative Exotic Richness	Intercept	0.131	0.408	1621	0.321	0.749	
	N addition	0.739	0.444	798	1.666	0.096	
	P addition	0.512	0.444	798	1.153	0.249	
	K & Micronut. Addition	-0.325	0.441	798	-0.737	0.461	
	N*P	-0.547	0.632	798	-0.865	0.387	
	N*K	1.011	0.630	798	1.606	0.109	
	P*K	0.101	0.630	798	0.160	0.873	
	N*P*K	0.033	0.892	798	0.037	0.971	
Native Cover	Intercept	3.327	1.998	1621	1.665	0.096	
	N addition	-1.205	1.132	798	-1.064	0.288	
	P addition	-2.004	1.132	798	-1.770	0.077	
	K & Micronut. Addition	-1.066	1.126	798	-0.947	0.344	
	N*P	1.443	1.611	798	0.896	0.371	
	N*K	0.940	1.607	798	0.585	0.559	
	P*K	2.016	1.607	798	1.255	0.210	
	N*P*K	-3.622	2.276	798	-1.591	0.112	
Exotic Cover	Intercept	-1.925	1.486	1621	-1.295	0.196	
	N addition	3.778	1.109	798	3.406	0.001	***
	P addition	2.514	1.110	798	2.265	0.024	*
	K & Micronut. Addition	-0.143	1.104	798	-0.130	0.897	
	N*P	-1.024	1.579	798	-0.648	0.517	
	N*K	0.328	1.575	798	0.208	0.835	
	P*K	-0.064	1.575	798	-0.041	0.968	
	N*P*K	0.227	2.231	798	0.102	0.919	
Relative Exotic Cover	Intercept	-0.869	0.824	1621	-1.055	0.292	
	N addition	1.812	0.624	798	2.906	0.004	**
	P addition	1.353	0.624	798	2.168	0.030	*

K & Micronut. Addition	0.071	0.621	798	0.115	0.909
N*P	-0.600	0.888	798	-0.676	0.499
N*K	0.029	0.886	798	0.033	0.974
P*K	-0.656	0.886	798	-0.741	0.459
N*P*K	0.922	1.255	798	0.735	0.463

Supplementary Table 7. Contributions of authors in the following categories: Coordinated Network (e.g. designed experiments, coordinated network data collection and compilation, and garnered funding), Developed and framed research (contributed substantially to framing questions for this specific study), Collected Data, Analyzed data (e.g. generated statistical or mathematical models, figures, tables, maps, diagrams), Contributed to data analyses (e.g. provided comments and suggestions on analyses), Wrote the paper (e.g. wrote the majority of at least one section of the paper), Contributed to paper writing (e.g. provided comments and suggestions on manuscript).

Name	Coordinated Network	Developed and framed research	Collected Data	Analyzed data	Contributed to data analyses	Wrote paper	Contributed to paper writing	Site-specific Acknowledgments
Seabloom, Eric	X	X	X	X	X	X	X	
Borer, Elizabeth	X	X	X		X		X	
Buckley, Yvonne			X	X	X		X	
Cleland, Elsa E.			X		X		X	
Davies, Kendi			X		X		X	
Firn, Jennifer			X		X		X	
Harpole, W. Stanley	X		X		X		X	
Hautier, Yann			X		X		X	
Lind, Eric	X		X	X	X		X	
MacDougall, Andrew			X		X		X	
Orrock, John L.			X		X		X	
Prober, Suzanne M			X		X		X	
Adler, Peter			X				X	
Anderson, T. Michael			X					
Bakker, Jonathan D.			X				X	
Biederman, Lori A.			X					
Blumenthal, Dana M.			X				X	
Brown, Cynthia S.			X				X	
Brudvig, Lars			X				X	
Cadotte, Marc			X					
Chu, Chengjin			X					Fundamental Research Funds for the Central Universities (lzujbky-2013-k15) and the Program for New Century Excellent Talents in University (NCET-12-0248)

Cottingham, Kathryn L.		X			
Crawley, Michael J.		X	X		
Damschen, Ellen I.		X			
Dantonio, Carla M		X		X	
DeCrappeo, Nicole M.		X			
Du, Guozhen		X			
Fay, Philip A		X		X	
Frater, Paul		X		X	
Gruner, Daniel S.		X		X	
Hagenah, Nicole		X		X	
Hector, Andy		X		X	
Hillebrand, Helmut		X			
Hofmockel, Kirsten S.		X		X	
Humphries, Hope C.		X			
Jin, Virginia L.		X		X	
Kay, Adam		X		X	
Kirkman, Kevin P		X		X	
Klein, Julia A.		X			
Knops, Johannes M. H.		X		X	
La Pierre, Kimberly J.		X		X	
Ladwig, Laura		X			
Lambrinos, John G.		X			
Li, Qi		X			
Li, Wei		X			
Marushia, Robin		X		X	
McCulley, Rebecca L.		X		X	Jim A. Nelson and A.E. Carlisle and the Kentucky Agricultural Experiment Station
Melbourne, Brett		X			
Mitchell, Charles		X		X	
Moore, Joslin L.		X		X	
Morgan, John		X		X	
Mortensen, Brent		X		X	
O'Halloran, Lydia R.	X	X			

Pyke, David	X		
Risch, Anita C.	X	X	
Sankaran, Mahesh	X		
Schuetz, Martin	X	X	
Simonsen, Anna	X		
Smith, Melinda	X		
Stevens, Carly J.	X	X	R. Rhodes
Sullivan, Lauren	X	X	
Wolkovich, Elizabeth M.	X	X	
Wragg, Peter D.	X	X	
Wright, Justin	X		
Yang, Louie	X	X	
